# Experimental investigation on tensile strength and impact strength of palmyra palm leaf stalk – Sisal fiber reinforced polymer hybrid composite

**DOI:** 10.1016/j.heliyon.2024.e39555

**Published:** 2024-10-18

**Authors:** Adugnaw Ayalew Bekele, Haymanot Takele Mekonnen, Belete Sirahbizu Yigezu, Abyot Yassab Nega

**Affiliations:** a Department of Mechanical Engineering, School of Mechanical and Chemical Engineering, Woldia Institute of Technology, Woldia University, Woldia, Ethiopia; b Department of Electrical and Computer Engineering, Institute of Technology, Debre Markos University, Debre Markos, Ethiopia; c Department of Mechanical Engineering, College of Electrical and Mechanical Engineering, Addis Ababa Science and Technology University, Addis Ababa, Ethiopia

**Keywords:** Handy lay-up, Hybrid fiber, Mechanical properties. unsaturated polyester resin

## Abstract

Natural fiber-reinforced polymer composites are the most widely used materials and preferable in terms of biodegradability, cost production, recyclability, and low density. The main aim of this study is to conduct an experimental investigation on tensile strength and impact strength of palmyra palm leaf stalk fiber (PLSF) and sisal fiber reinforced polymer hybrid composite. The composite material was fabricated using hand lay-up techniques. The working parameters are mass fraction ratio of PLSF/sisal fiber and volume fiber fraction with the matrix. Tensile strength and impact energy resistance tests were experimentally conducted according to the ASTM standard dimensions. The results revealed that the addition of sisal fiber to PLSF enhanced the tensile strength by 12.850 %, 26.540 %, and 30.630 % respectively compared to pure Palmyra palm leaf stalk fiber reinforced composite (PPFRC). Whereas, the addition of PLSF to sisal fiber improved the impact of energy by 20.980 %, 13.610 %, and 11.880 % compared to pure sisal fiber reinforced composite (PSFRC). The tensile strength with 20 % fiber volume fraction is improved by 53.996 % and 12.188 % compared to 10 % and 15 % of fiber respectively. The impact strength was also enhanced by 24.931 % and 10.030 % compared to 10 % and 15 % of volume fiber fraction respectively. The tensile strength and impact energy of the treated fiber composite increased by 62.243 % and 22.478 % respectively compared to the untreated hybrid Palmyra palm leaf stalk and sisal hybrid fiber reinforced composite (UHPSFRC). Generally, the HPSFRC-2 (Palmyra palm leaf stalk/sisal fiber) (P/S ratio 50/50 % ratio with 20/80 % ratio of fiber/matric percentage reinforced polymer hybrid composite) has good tensile strength and impact energy. Therefore, the mechanical property of the (Palm/Sisal) hybrid composite can be used for the manufacturing of the automotive interior parts like door panel, dash board, seat back, and automotive roof.

## 1 Introduction

Nowadays, the world needs materials that have the properties of biodegradability, renewability, environmentally friendly, less cost, and light in weight to save power [1]. However, the substitution of locally available materials which are compatible with the mechanical properties of composite materials is limited. In addition to these, synthetic fibers can also lead the company to extra expensive and environmental pollution during the disposal of waste materials. These properties can be achieved using natural fibers by studying mechanical properties experimentally. Natural fibers are a substitute for synthetic fiber for use as reinforcements in composite materials due to hazardous, rigidity, and are expensive with depleting resources [2]. Natural fibers are offered many advantages through different properties. These are: low density, biodegradable, recyclability, low cost per unit volume, sustainability in production, and high strength to weight ratio. Among natural fibers, palmyra palm leaf-stalk and sisal fiber have been considered and used in the automobile industry.

Hybrid natural fiber composite materials are one of the emerging material sciences fields which mainly need for the sustainable development of worldwide in different engineering applications. A single fiber does not hold all the necessary mechanical properties such as tensile strength, flexural strength, impact strength, elongation at the failure point, etc. Some natural fibers have greater tensile strength and flexural strength but are weak in impact strength and elongation at the breakdown. However, some natural fibers display superior properties of impact strength and elongation at break, but may have weak performance in tensile and flexural strength. That is why the investigators conducted and analyzed composites using two or more natural fibers with resin matrix [3]. The behavior of hybrid composites is the sum of individual parts considered in which there is the stability between the essential advantages and disadvantages [4,5]. Therefore, numeral fibers which are used as a reinforcement in a single matrix phase are hybrid composite [6]. D. Shanmugam and Thiruchitrambalam (2013) [[7], [8]] have studied the static and dynamic properties of alkaline treated unidirectional continuous palmyra palm leaf stalk fiber/jute fiber reinforced hybrid polyester composites. They concluded that the addition of jute fiber improved the tensile and flexural strength of hybrid composite as compared to the palmyra palm leaf stalk fiber-reinforced composite. But the impact strength of the hybrid composite observed less property than palmyra palm leaf stalk fiber-reinforced composite.

G. H. Staab (2015) [9] investigates the maximum mechanical strength obtained through continuous and unidirectional reinforcement composites, rather than bulk and discontinuous materials. Continuous unidirectional fiber composites are used for this study.

Goriparthi et al. [10] suggested that a high amount of treatment material had a disadvantage while treating natural fibers. The chemical treatment, such as the alkali concentration and the period of the fiber soaking, is primarily responsible for the improvement of mechanical and physical properties. When treated with 5 % weight NaOH, the jute/coir/polymer hybrid composite exhibits superior qualities; when the chemical concentration increased to 10 % of NaOH, the hybrid composite exhibits lower properties. Therefore, even at minimized load transfer for various applications, the fiber becomes brittle, weak, and inadequate due to the increased removal of hemicellulose and lignin. Generally, the higher NaOH solution treatment decreases the percentage of fiber-cellulose content that leads to decreases in the mechanical properties of a single fiber.

This study mainly focuses on the experimental investigation of mechanical properties of Palmyra palm leaf stalk fiber and sisal fiber with unsaturated polyester resin polymer. The extracted fiber is treated by an alkaline treatment processed with a 5 % concentration of NaOH solution. The study parameters focus on the different mass percentages of Palmyra palm leaf stalk/sisal (P/S) fiber and volume fraction (fiber/matrix) of hybrid composites. In this study, PLSF/sisal fiber reinforced hybrid composite samples were fabricated using a handy lay-up mold technique to improve the mechanical properties of those who are lacking one another. The study was carried out on different mass fractions of individual fiber ratio and volume fiber fraction. According to the previous investigations, fiber orientation was uniaxial rather than random oriented. Therefore, in this fiber orientation, the maximum mechanical properties of hybrid composites are recorded. The current study deals with the sisal and palmyra palm-leaf stalk hybrid composite with the application of automotive interior parts like door panels.

## 2 Materials and methods

### 2.1 Fiber preparation

#### 2.1.1 Extraction of palmyra palm leaf stalk fiber

Palmyra palm leaf-stalk fiber was extracted from the palmyra palm tree through a manual extraction method. Firstly, the free aged and old leaf stalks were collected from the palmyra palm tree. Then the thorns and skin found at the edge of palmyra palm leaf-stalks were cut off using a knife. Each stalk was cut into strips and retted in water for 40 days to suck the tap water and become wet. Then the retted stalks were beaten using a wooden hammer. The individual fiber was separated manually and cleaned with a neat cloth to remove external impurities. Lastly, the fiber was washed using distilled water and dried in exposed sunlight for three days.

#### 2.1.2 Extraction of sisal fiber

Firstly, the sisal leaves were cut from the mature sisal plant. Then make a shortened strip of the leaf by trimming both ends for ease of fiber extraction. The outer layer of the leaf stalk was peeled using a knife manually. The fiber was washed to separate a single fiber from stacked fiber and dried in open sunlight for four days.

### 2.2 Alkaline treatment

The dried fibers were immersed with 5 % NaOH solution using distilled water for 24 h. Next, the fibers were washed with tap water to remove the lignin, wax, hemicellulose, and other impurities. Finally, the removed fibers were dried to expose sunlight for three days.

### 2.3 Preparation of composite

The PPLSF used in the hybrid composite is mixed with sisal fiber homogeneously and this mixture of fiber is combined with matrix randomly. Finally, the hybrid composite is formed. Therefore, palmyra palm leaf stalk and sisal fiber were mixed homogeneously. The fibers were arranged using continuous unidirectional orientation. The matrix material was prepared using unsaturated polyester resin mixed with catalyst or hardener. The amount of hardener added for composite preparation was 2 % of the total matrix [11,12]. The composites were fabricated using the hand lay-up method with a wooden frame mold length of 260 mm; a width of 150 mm thickness of 3 mm. The load application process for curing composite was also carried out. After the curing composites were removed from the mold and the specimen preparation using a grinding cutting machine was operated according to the ASTM standards dimension. The composites were manufactured by changing the volume fiber fraction of PLF/sisal fiber with 20 % of total volume fiber fraction. The second parameter design was manufactured varying from the fiber/matrix ratio (10/90 %, 15/85 %, and 20/80 %) with constant fiber/fiber ratio (50/50 % of Palmyra palm leaf stalk/sisal fiber). The composition was fabricated with different mass fractions of palmyra leaf stalk fiber and sisal fiber at a 20 % constant matrix volume fraction.

Composites samples are designated by HPSFRC-1, HPSFRC-2, HPSFRC-3, HPSFRC-4, and HPSFRC-5. The HPSFRC (1, 2, and 3) were fabricated from 20 % of total fiber volume fractions with the ratio of palm/sisal in percent 75 %/25 %, 50 %/50 %, and 25 %/75 % respectively. However, the HPSFRC of (4 and 5) composites were fabricated with the same percentage of palm/sisal (50 %/50 %) for 10 % and 15 % of volume fiber fraction with 90 % and 85 % of matrix volume fraction respectively.

### 2.4 Mechanical properties

#### 2.4.1 Tensile strength

**Fig. 1:**
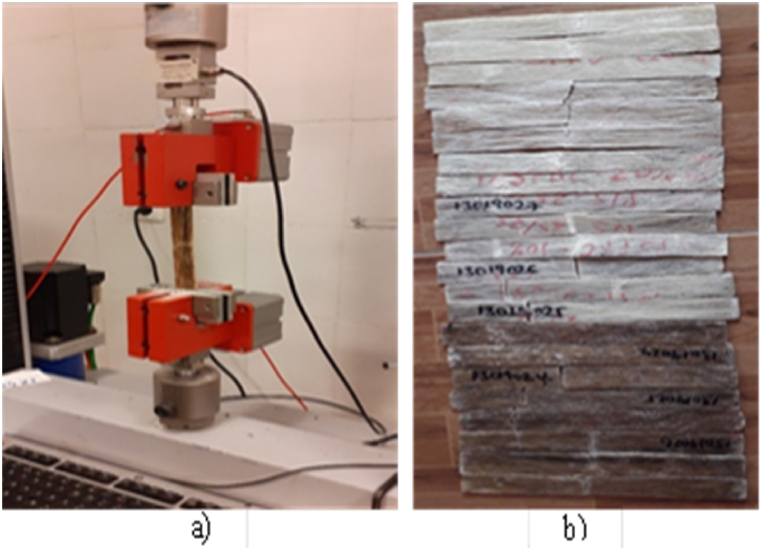
a) Tensile test under tensile testing machine b) fractured specimen after tensile load.

The standard specific dimension of tensile strength test was conducted as per the ASTM: D3039 specimen dimension of 250 mm in length, 25 mm in width, and 3 mm thickness. The test was carried out through a uniaxial load by applying over both ends of the sample specimen at a crosshead speed of 10 mm/min as shown in Fig. 1.

Fig. 1a shows the universal testing machine under tensile test operating and Fig. 1b show the fractured specimens after tensile tests is carried out.

#### 2.4.2 Impact strength

**Fig. 2:**
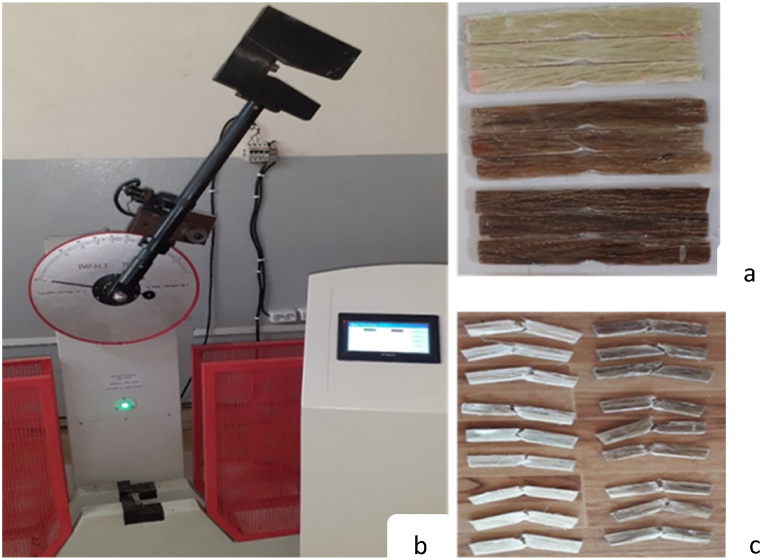
a) Impact test specimens b) Impact testing machine under operation. c) Test specimen after impact test.

The Charpy impact test was operated using JBS -500B Touch screen digital display Charpy impact testing machine as shown in Fig. 2. The specimen size of the fabricated sample composite was prepared as per ASTM: D256 standard specimen dimension of 63.5 mm in length, 12.5 mm width and 3 mm in thickness. At the center of the specimen, a ‘V’ notch of 2.54 mm depth and 45^0^ notch angles was formed.

Fig. 2a shows the impact test specimen before testing operation; Fig. 2b shows the impact testing machine under impact test operation with JBS -500B model, and Fig. 2c shows the fractured impact test specimens after impact testing operation is caried out.

## 3 Results and discussion

The present study discussed the experimental investigation of mechanical properties through tensile and impact strength of PLSF and sisal hybrid fiber. The HPSFRC was fabricated by 10 %, 15 %, and 20 % of hybrid volume fiber fractions and with 90 %, 85 %, and 80 % of the polyester matrix of volume fractions respectively. This hybrid fiber has a constant value in the mass fraction with 50 % of palmyra palm leaf stalk fiber and 50 % of sisal fiber. In the other case, the composite was manufactured based on varying mass fractions of PPLSF and SF. The PPLSF/SF (P/S (%)) of the composite was fabricated with different mass fractions, including 100/0, 75/25, 50/50, 25/75, 0/100. However, the total value in hybrid fiber of volume fraction was 20 % of fiber with 80 % of the matrix in volume fraction.

### 3.1 Tensile strength

The experiment was evaluated by using a universal testing machine. The average tensile strength value of the three specimens for each composite is tabulated in Table 1. The relevant results of tensile strength test data are presented with corresponding strain, stress, force at break, force at the peak and elongation in Table 2.

**Table 1:** Volume content of fiber and matrix for composite fabrication.

Designed parameter	Palmyra palm leaf stalk/sisal (P/S) (%)	Fiber/Matrix (%)
PPFRC	100/0	20/80
PSFRC	0/100	20/80
HPSFRC-1	75/25	20/80
HPSFRC-2	50/50	20/80
HPSFRC-3	25/75	20/80
HPSFRC-4	50/50	10/90
HPSFRC-5	50/50	15/85
UHPSFRC	50/50	20/80

Where: P – Palmyra palm leaf stalk fiber.

S – Sisal fiber.

PPFRC - Pure Palmyra Palm Leaf Stalk Fiber Reinforced Composite.

PSFRC - Pure Sisal Fiber Reinforced Composite.

HPSFRC-1 – Palmyra palm leaf stalk – Sisal Fiber Reinforced Hybrid Composite with P/S of 75/25 % and Fiber/Matrix of 20/80 %.

HPSFRC-2 - Palmyra palm leaf stalk – Sisal Fiber Reinforced Hybrid Composite with P/S ratio of 50/50 % and Fiber/Matrix ratio of 20/80 %.

HPSFRC-3 - Palmyra palm leaf stalk – Sisal Fiber Reinforced Hybrid Composite with P/S ratio of 25/75 % and Fiber/Matrix ratio of 20/80 %.

HPSFRC-4 - Palmyra palm leaf stalk – Sisal Fiber Reinforced Hybrid Composite with P/S ratio of 50/50 % and Fiber/Matrix ratio of 10/90 %.

HPSFRC-5 - Palmyra palm leaf stalk – Sisal Fiber Reinforced Hybrid Composite with P/S ratio of 50/50 % and Fiber/Matrix ratio of 15/85 %.

UHPSFRC – Untreated Palmyra palm leaf stalk – Sisal Fiber Reinforced Hybrid Composite with P/S of 50/50 % and Fiber/Matrix ratio of 20/80 %.

**Table 2:** Tensile strength test results of different designed parameters.

Designed parameter	Test No.	Strain @ Break (%)	Stress @ Break (N/mm^2^)	Force @ Break (N)	Elong. @ Break (mm)	Force @ Peak (N)
**PPFRC**	Mean	3.985	24.878	1865.867	5.977	2008.767
	S.D.	0.178	2.579	193.441	0.267	157.296
	C. of V.	4.461	10.367	10.367	4.461	7.83
**PSFRC**	**Mean**	**4.131**	**37.366**	**2802.433**	**6.197**	**2817.7**
	S.D.	0.943	7.916	593.676	1.414	610.315
	C. of V.	22.825	21.184	21.184	22.825	21.66
**HPSFRC-1**	**Mean**	**2.879**	**28.075**	**2105.6**	**4.318**	**2121.367**
	S.D.	0.446	7.352	551.39	0.669	545.818
	C. of V.	15.502	26.187	26.187	15.502	25.73
**HPSFRC-2**	**Mean**	**3.022**	**31.48**	**2361.0**	**4.533**	**2478.6**
	S.D.	0.386	6.061	454.587	0.579	377.198
	C. of V.	12.779	19.254	19.254	12.779	15.218
**HPSFRC-3**	**Mean**	**3.799**	**32.499**	**2437.475**	**5.670**	**2528.950**
	S.D.	0.67	6.812	510.921	1.005	460.632
	C. of V.	17.633	20.961	20.961	17.633	18.214
**HPSFRC-4**	**Mean**	**2.773**	**20.442**	**1533.133**	**4.159**	**1576.967**
	S.D.	0.206	3.394	254.533	0.309	196.254
	C. of V.	7.432	16.602	16.602	7.432	12.445
**HPSFRC-5**	**Mean**	**2.964**	**28.06**	**2104.467**	**4.446**	**2150.933**
	S.D.	0.443	2.057	154.27	0.665	234.752
	C. of V.	14.958	7.331	7.331	14.958	10.914
**UHPSFRC**	**Mean**	**2.049**	**19.403**	**1455.2**	**3.074**	**1488.8**
	S.D.	0.747	9.942	745.654	1.12	738.584
	C. of V.	36.454	51.241	51.241	36.454	49.609

Where.

Mean - average testing value.

S.D – standard deviation.

C.of V. – coefficient of variation.

**Fig. 3:**
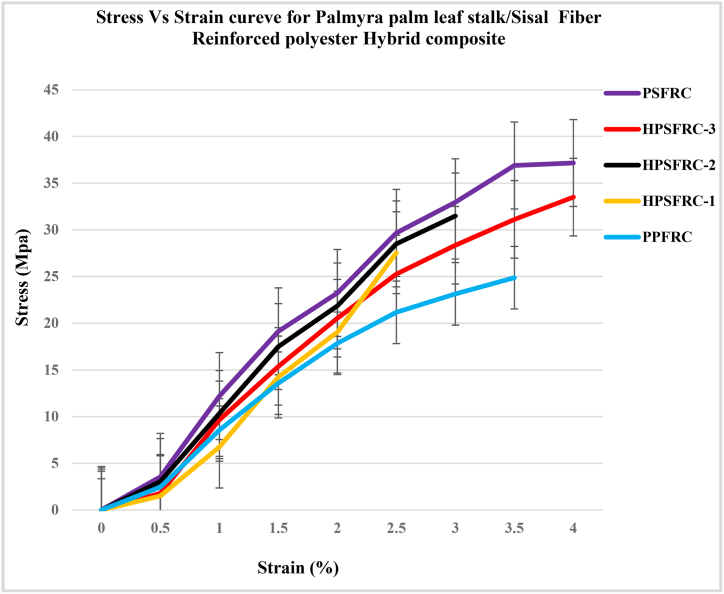
Stress – Strain curve of PPFRC, PSFRC and different Hybrid Palm leaf stalk/Sisal Reinforced polyester composite.

In the above Fig. 3 it is observed that the pure sisal fiber-reinforced composite shows higher tensile strength than the pure Palmyra palm leaf stalk reinforced fiber composite. Due to the presence of sisal fiber, the HPSFRC exhibits higher tensile strength than the tensile strength of PPFRC but lowers tensile strength than PSFRC. The tensile strength of the palm/sisal with 75/25(%), 50/50 (%), and 25/75(%) ratio was 28.075 Mpa, 31.48 Mpa, and 34.158 Mpa respectively. Therefore, the tensile strength of HPSFRC was increased by 12.85 %, 26.54 %, and 37.3 % respectively compared to pure Palmyra palm leaf stalk fiber-reinforced composite.

This indicates that the sisal fiber-reinforced composite has a higher capacity to resist the tensile load than the Palmyra palm leaf stalk fiber-reinforced composite. Different designations of pure and hybrid fiber reinforced composites are shown in figures below taken from the individual sample testing tensile operations.

**Fig. 4:**
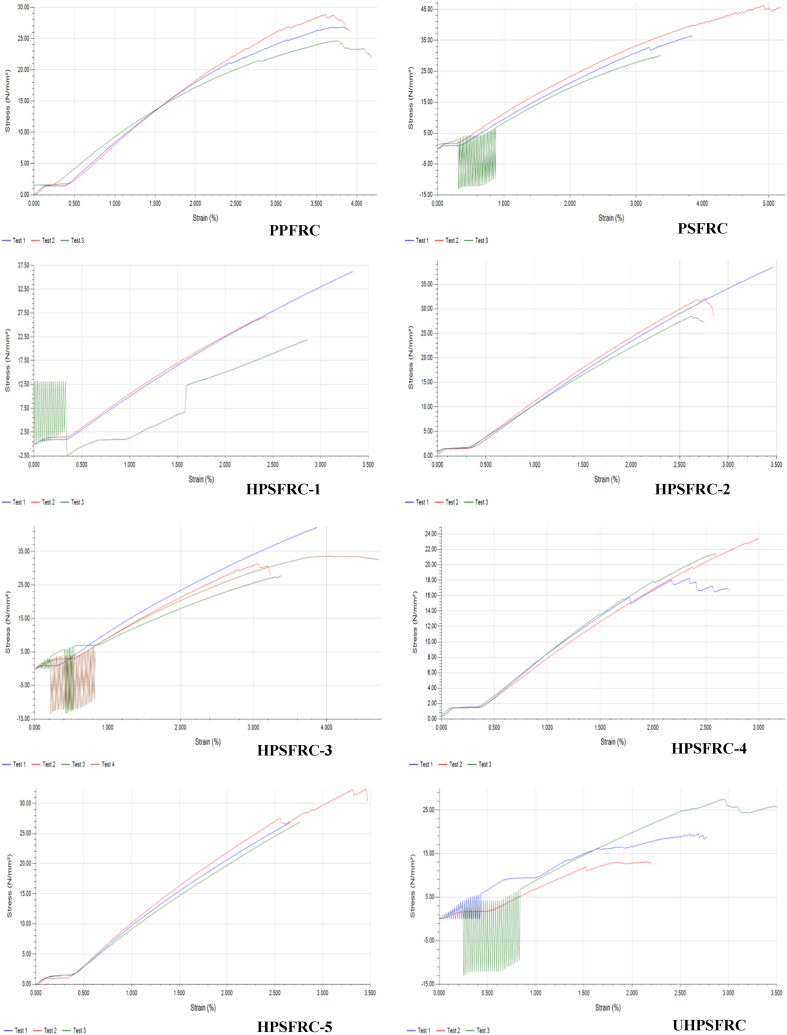
Tensile Stress vs strain for different designed parameters.

Fig. 4 Tensile Stress vs strain for different designed parameters shows the elasticity and plasticity region for pure palmyra palm leaf stalk fiber reinforced composite (PPFRC), pure sisal fiber-reinforced composite (PSFRC), hybrid composites (HPSFRC 1 - HPSFRC 5), and untreated hybrid composite (UHPSFRC). From the curve shown in the figures, the PSFRC curve has better elasticity properties compared to PPFRC and other hybrid composites. Therefore, the percentage of elongation as increasing the applied load shows a higher value. However, the elongation property of pure sisal fiber-reinforced composite has a higher value compared to pure Palmyra palm leaf stalk fiber-reinforced composite. Therefore, the PSFRC ductility property due to the ultimate strength and fracture points of the composite sample are far apart. However, the PPFRC is brittle as a result of the ultimate strength and fracture point is close to each other compared to the PSFRC.

Finally, the curve shows that the tensile strength of alkaline treated composites has better tensile strength property than the untreated hybrid composite. Due to the presence of impurities on the untreated fiber, the load-carrying capacity for the tensile strength test is decreased. As a result of better surface finishing fiber through alkaline treatment, fiber and matrix bond interaction is a better property.

### 3.2 Impact strength

Three specimens were tested and the average values were reported. The results found in the test are included in Table 3. Based on the outcome results, the properties of impact energy are related to the bond between palmyra palm leaf-stalk fiber and sisal fiber with the matrix material.

**Table 3:** Impact energy and toughness for different hybrid HPSFRC test results.

Designed parameter	Impact Energy (J)	Toughness (J/cm^2^)
PPFRC	53.72	8.91
PSFRC	43.93	7.28
UHPSFRC	40.75	6.75
HPSFRC-1	53.15	8.81
HPSFRC-2	49.91	8.27
HPSFRC-3	49.15	8.15
HPSFRC-4	39.95	6.62
HPSFRC-5	45.36	7.46

Table 2 shows that PPFRC has recorded the maximum impact energy and toughness results compared to PSFRC and other HPSFRC. Hybrid composite HPSFRC-3, HPSFRC-2 and HPSFRC-1 have better impact energy results compared to PSFRC. Therefore, addition of palmyra palm leaf-stalk fiber to the sisal fiber enhances the impact-absorbing energy of the hybrid composite. The impact energy of the composite has a positive effect on the alkaline surface treatment of palmyra palm leaf stalk and sisal fiber.

### 3.3 Comparison with the previously published works

#### 3.3.1 Comparison of tensile strength

The tensile strength of palmyra palm leaf stalk/sisal fiber reinforced the polyester hybrid composite is comparable to other natural fiber reinforced polymer composites. Table 4 shows that the tensile strength of the present study was improved by 3.79 %, 11.00 %, and 22.20 % of Banana/epoxy (kenaf/flax)/Polyester, and date palm/epoxy respectively. From the literature, it can be understood that the tensile strength of hybridization can be enhanced from a single fiber reinforced composites.

**Table 4:** Comparison of tensile strength for various fiber reinforced composites.

Comparison of tensile strength from previously published works						
Fiber/Matrix Material	Composite fabrication Method	Fiber /Matrix (%)	Fiber/Fiber (%)	Fiber orientation	Tensile strength (Mpa)	Reference
**(PLSF/Sisal)/Polyester**	**Handy lay-up**	**20/80**	**50/50**	**Unidirectional Continuous**	**31.48**	**Present study**
Sugar Palm yarn/Polyester	Handy lay-up	20/80	–	Unidirectional continuous	23.92	S. Malaysiana (2018) [11]
Kenaf/Flax/Polyester	Compression Molding	40/60	50/50	Random	28.36	Sathish et al. (2017) [12]
(Palmyra/Glass)/Rooflite	Compression Molding	31/69	50/50	Random	26.3	R. Velmurugan and V. Manikandan (2007) [13]
Sisal/Epoxy	Compression Molding	20/80	–	Unidirectional Continuous	38.83	M. Kumaresan et al. (2015) [14]
Date Palm/Epoxy	Spray-up	50/50	–	Chopped	25.76	N. Saba et al. (2019) [15]
(Sisal/Coir)/Epoxy	Handy lay-up	40/60	50/50	Chopped	30	T. Nadu and T. Nadu (2014) [16]
(Coire/Silk)/Polyester	Handy layup	20/80	–	Random	16.144	Z. Dashtizadeh et al. (2017) [17]
Banana/Epoxy	Handy lay-up	30/70	–	Random	30.33	N. Venkateshwaran (2013) [18]

#### 3.3.2 Comparison of impact strength

The present study of impact strength for Palmyra palm leaf stalk was comparable to coir/jute, curacao/glass, Palmyra/glass, PLSF/glass/coconut, banana/sisal, and Palmyra/jute of hybrid composite as discussed in Table 5. Therefore, from the discussion, it can be concluded that the toughness property of (PLSF/Sisal) hybrid fiber reinforced can be used in place of other natural fiber/natural fiber and natural fiber/glass hybrid fiber properties. Palmyra palm leaf-stalk fiber exhibits relatively high impact strength properties and elongations compared to other fibers like sisal, jute, flax, banana, and palm fruit.

**Table 5:** Comparison of toughness for various fiber reinforced composites.

Comparison of toughness from previously published works						
Fiber/Matrix Material	Composite fabrication Method	Fiber/Matrix (%)	Fiber/Fiber (%)	Fiber orientation	Toughness (J/cm^2^)	Reference
**(PLSF/Sisal)/Polyester**	**Handy layup**	**20/80**	**50/50**	**Unidirectional Continuous**	**8.27**	**Present study**
(Coir/Jute/Polypropylene	Compression	20/80	20/80	Unidirectional continuous	3.0	A. H. Khan et al. (2010) [13]
(Curaua/Glass)/Polyester	Handy layup	30/70	50/50	Chopped	10.69	J. H. S. Almeida Júnior (2012) [14]
Palmyra/glass	Handy layup	55	49/6	Random	5.01	R. Velmurugan and V. Manikandan (2007) [15]
(PLSF/E-glass/Coconut)/Polyester	Handy layup	30/70	10/15/5	Random	9.6	E. R. Dhas and P. Pradeep (2017) [12]
(Banana/Sisal)/Polyester	Compression Molding	40/60	50/50	Bi-layer Random	4.3	P. H. P. O (2010) [16]
(Palmyra/jute)/Polyester	Handy layup and Compression Molding	30/70	75/25	Unidirectional Continuous	3.5	D. Shanmugam and M. Thiruchitrambalam (2013) [7]
Bamboo/Polyester	Handy layup	20/80	–	Random	4.51	H. Banga (2015) [17]

## 4 Conclusion

In this study, palmyra palm leaf stalk fiber and sisal fiber were used as reinforcement material. Polyester resin provides the necessary binding and matrix material for the fabrication of composite material. Sodium hydroxide (NaOH) of the alkaline solution is used to improve the interfacial interaction of the matrix, reinforcement fiber, and adhesion properties of the hybrid composite. The palmyra palm leaf stalk-sisal fiber reinforced hybrid composite was fabricated using different parameters. Based on the mass fraction and volume fiber fraction with fiber/matrix (P/S) ratio was fabricated. The mass fraction for P/S includes 100/0, 75/25, 50/50, 25/75, and 0/100 in percentage at a constant fiber/matrix of 20/80 ratio. The volume fraction of fiber/volume includes 10/90, 15/85, 20/80. This was fabricated in the present study at a constant P/S of 50/50 (%) ratio. The characterization of mechanical properties was carried out through tensile strength and impact energy.

The tensile strength result based on the mass fraction of P/S ratio was found that the tensile strength test of the hybrid composite was increased by increasing the sisal fiber. Based on the fiber/matrix volume fraction, which includes 10/90, 15/85, 20/80 was recorded 20.442 Mpa, 28.06 Mpa, and 31.49 Mpa respectively. The maximum tensile strength is observed at 20/80 of volume fraction. Based on the mass fraction, the toughness value is 8.81 J/cm2, 8.27 J/cm2, and 8.15 J/cm2 respectively. As a result, the impact of energy and toughness value decreased with increasing the sisal fiber. Therefore, the results found that sisal fiber has higher tensile strength and lower impact energy than single palmyra palm leaf stalk fiber. The treated fiber reinforced composites have better tensile strength and impact strength than the untreated fiber composite due to the removal of lignin, wax, hemicellulose, and other impurities which decrease the properties of fiber-matrix bond interaction.

Generally, this study result concludes that the hybridization of palmyra palm leaf stalk – sisal fiber reinforced polymer hybrid composites is a very effective way of improving the property of tensile strength and impact energy which can be used in automobile parts such as interior door panels, pillars, backrests, and dashboards.

## CRediT authorship contribution statement

**Adugnaw Ayalew Bekele:** Writing – review & editing, Writing – original draft, Visualization, Validation, Supervision, Software, Resources, Project administration, Methodology, Investigation, Funding acquisition, Formal analysis, Data curation, Conceptualization. **Haymanot Takele Mekonnen:** Writing – review & editing, Software, Resources, Methodology, Investigation, Formal analysis, Data curation, Conceptualization. **Belete Sirahbizu Yigezu:** Visualization, Data curation. **Abyot Yassab Nega:** Writing – review & editing.

## Declaration of competing interest

The authors declare that they have no known competing financial interests or personal relationships that could have appeared to influence the work reported in this paper.

## References

[1] Betelie A.A., Sinclair A.N., Kortschot M., Li Y. (2019). Mechanical properties of sisal-epoxy composites as functions of fiber-to-epoxy ratio.

[2] Jawaid M., Abdul Khalil H.P.S., Abu Bakar A. (2011). Woven hybrid composites: tensile and flexural properties of oil palm-woven jute fibres based epoxy composites. Mater. Sci. Eng. A.

[3] Rahman M.Z. (2003).

[4] Sapuan S.M., Bin Yusoff N. (2015).

[5] Pappu A., Pickering K.L., Thakur V.K. (2019). Manufacturing and characterization of sustainable hybrid composites using sisal and hemp fibres as reinforcement of poly (lactic acid) via injection moulding. Ind. Crops Prod..

[6] Jacob M., Thomas S., Varughese K.T. (2004). Mechanical properties of sisal/oil palm hybrid fiber reinforced natural rubber composites. Compos. Sci. Technol..

[7] Shanmugam D., Thiruchitrambalam M. (2013). Static and dynamic mechanical properties of alkali treated unidirectional continuous Palmyra Palm Leaf Stalk Fiber/jute fiber reinforced hybrid polyester composites. Mater. Des..

[8] Akash, Gupta N. S. Venkatesha, Sreenivas Rao K.V. (2018). An experimental study on sisal/hemp fiber reinforced hybrid composites. Mater. Today Proc..

[9] Staab G.H. (2015).

[10] Goriparthi B.K., Suman K.N.S., Mohan Rao N. (2012). Effect of fiber surface treatments on mechanical and abrasive wear performance of polylactide/jute composites. Compos. Part A Appl. Sci. Manuf..

[11] Xue D., Hu H. (2013). Mechanical properties of biaxial weft-knitted flax composites. Mater. Des..

[12] Dhas E.R., Pradeep P. (2017). Mechanical property evaluation of palm/glass sandwiched fiber reinforced polymer composite in comparison with few natural composites. IOP Conf. Ser. Mater. Sci. Eng..

[13] Khan A.H., Hossain M.A., Khan M.A., Khan R.A. (2010). J. Composer. Mater..

[14] Almeida Júnior J.H.S., Ornaghi Júnior H.L., Amico S.C., Amado F.D.R. (2012). Study of hybrid intralaminate curaua/glass composites. Mater. Des..

[15] Velmurugan R., Manikandan V. (2007).

[16] P. O P.H. (2010). “Mechanical Performance of Short Banana/Sisal Hybrid Fiber Reinforced Polyester Composites,”.

[17] Banga H., Singh V.K., Choudhary S.K. (2015). Fabrication and study of mechanical properties of bamboo fibre reinforced bio-composites. Innovat. Syst. Des. Eng..

